# 1-(3-Bromo­prop­yl)-4-(2-pyrid­yl)-1*H*-1,2,3-triazole

**DOI:** 10.1107/S1600536809012148

**Published:** 2009-04-08

**Authors:** James D. Crowley, Pauline H. Bandeen, Lyall R. Hanton

**Affiliations:** a Department of Chemistry, University of Otago, PO Box 56, Dunedin, New Zealand

## Abstract

In the structure of the title compound, C_10_H_11_BrN_4_, the plane of the substituted 1,2,3-triazole ring is tilted by 14.84 (10)° with respect to the mean plane of the pyridine ring. The pyridine and closest triazole N atoms adopt an *anti* arrangement which removes any lone pair–lone pair repulsions between the N atoms. This conformation is further stabilized by weak intermolecular C—H⋯N inter­actions. There are two mol­ecules in the unit cell, which form a centrosymmetric head-to-tail dimer. The dimers are stabilized through π–π inter­actions [centroid–centroid distance = 3.733 (4) Å and mean inter­planar distance = 3.806 (12) Å] between the substituted 1,2,3-triazole ring and the pyridine rings in adjacent mol­ecules. Each dimer inter­acts with two neighbouring dimers above and below, forming a slipped stack of dimers through the crystal. The 3-bromo­propyl chain sits over the pyridine ring of a neighbouring mol­ecule and the triazole rings of nearby mol­ecules are adjacent.

## Related literature

For details of the Cu(I)-catalysed 1,3-cyclo­addition of organic azides with terminal alkynes, see: Rostovtsev *et al.* (2002 ▶); Tornoe *et al.* (2002 ▶); Meldal & Tornoe (2008 ▶). For applications of pyridyl-functionalized 1,2,3-triazoles, see: Li & Flood (2008 ▶); Meudtner & Hecht (2008 ▶); Krivopalov & Shkurko (2005 ▶); Li *et al.* (2007 ▶); Richardson *et al.* (2008 ▶). For related structures, see Schweinfurth *et al.* (2008 ▶); Obata *et al.* (2008 ▶).
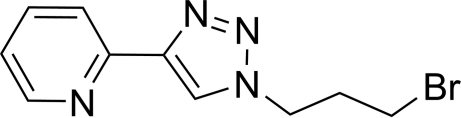


## Experimental

### 

#### Crystal data

C_10_H_11_BrN_4_
*M* *_r_* = 267.14
Triclinic, 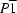

*a* = 5.658 (2) Å
*b* = 9.688 (4) Å
*c* = 10.191 (4) Å
α = 84.498 (3)°
β = 85.663 (2)°
γ = 83.854 (2)°
*V* = 551.6 (4) Å^3^
*Z* = 2
Mo *K*α radiation
μ = 3.70 mm^−1^
*T* = 90 K
0.53 × 0.23 × 0.11 mm

#### Data collection

Bruker APEXII CCD area-detector diffractometer
Absorption correction: multi-scan (*SADABS*; Bruker, 2004 ▶) *T*_min_ = 0.358, *T*_max_ = 0.66
8776 measured reflections
1879 independent reflections
1759 reflections with *I* > 2σ(*I*)
*R*_int_ = 0.043

#### Refinement

*R*[*F* ^2^ > 2σ(*F* ^2^)] = 0.027
*wR*(*F* ^2^) = 0.071
*S* = 0.97
1879 reflections
136 parameters
H-atom parameters constrained
Δρ_max_ = 0.64 e Å^−3^
Δρ_min_ = −0.61 e Å^−3^

### 

Data collection: *APEX2* (Bruker, 2004 ▶); cell refinement: *APEX2* and *SAINT* (Bruker, 2004 ▶); data reduction: *SAINT*; program(s) used to solve structure: *SIR97* (Altomare *et al.*, 1999 ▶); program(s) used to refine structure: *SHELXL97* (Sheldrick, 2008 ▶); molecular graphics: *ORTEP-3* (Farrugia, 1997 ▶) and *Mercury* (Bruno *et al.*, 2002 ▶); software used to prepare material for publication: *SHELXTL* (Sheldrick, 2008 ▶) and *enCIFer* (Allen *et al.*, 2004 ▶).

## Supplementary Material

Crystal structure: contains datablocks I, global. DOI: 10.1107/S1600536809012148/fj2204sup1.cif

Structure factors: contains datablocks I. DOI: 10.1107/S1600536809012148/fj2204Isup2.hkl

Additional supplementary materials:  crystallographic information; 3D view; checkCIF report

## Figures and Tables

**Table 1:** Hydrogen-bond geometry (Å, °)

*D*—H⋯*A*	*D*—H	H⋯*A*	*D*⋯*A*	*D*—H⋯*A*
C7—H7⋯N2^i^	0.93	2.62	3.449 (4)	149
C10—H10*B*⋯N1^ii^	0.97	2.51	3.450 (4)	164

Symmetry codes: (i) 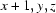; (ii) 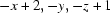.

**Fig. 1.**
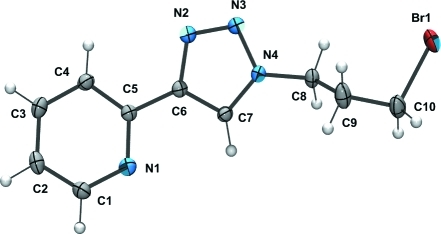
The molecular structure of compound 1, showing the atom numbering scheme. The thermal displacement ellipsoids are drawn at the 50% probability level.

**Fig. 2.**
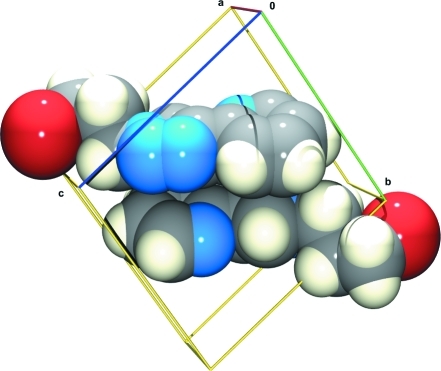
A spacefilling representation of the unit cell of 1 showing the head-to-tail stacking of the molecules.

**Fig. 3.**
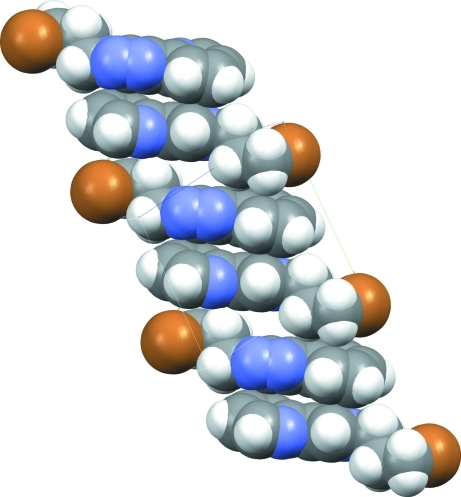
A spacefilling representation of the crystal packing present in 1, showing the slipped stacks of the dimers.

## supplementary crystallographic information

### Comment

The Cu(I)-catalyzed 1,3-cycloaddition of organic azides with terminal alkynes (the CuAAC reaction) (Rostovtsev *et al.* 2002; Tornoe *et al.* 2002) has quickly become an indispensable tool for functional molecule synthesis and renewed interest in the chemistry of functionalized 1,2,3-triazoles (Meldal & Tornoe, 2008). Because they are readily synthesized using the CuAAC reaction, pyridyl functionalized 1,2,3-triazoles have begun attracting significant attention in a range of areas including anion recognition (Li & Flood, 2008), stimuli responsive foldamers (Meudtner & Hecht, 2008), drug discovery (Krivopalov & Shkurko, 2005) and coordination chemistry (Li *et al.* 2007; Richardson *et al.*, 2008; Schweinfurth *et al.*, 2008; Obata *et al.*, 2008)

The molecular structure of the new triazole compound 1 is shown in Fig. 1. The molecule consists of an essentially co-planar (2-pyridyl) and 1,2,3-triazole units attached to a 3-bromopropyl chain, the plane of the substituted 1,2,3-triazole ring is tilted by 14.84 (10)° with respect to the mean plane of the pyridine ring system. As is commonly observed (Obata *et al.*, 2008; Schweinfurth *et al.*, 2008) N1 in the pyridine ring and N2 of the triazole ring adopt an anti arrangement which removes any lone pair-lone pair repulsions between the nitrogen atoms. Additionally, the anti conformation is stabilized by weak C—H···N interactions. There are two molecules of 1 in the unit cell which form a centrosymmetric head to tail dimer that is stabilized through a π-π interaction [centroid-centroid distance = 3.733 (4) Å, mean interplanar distance 3.806 (12) Å] between the substituted 1,2,3-triazole and the pyridine rings in adjacent molecules. In the extended crystal each dimer interacts with two neighbouring dimers above and below to form slipped stacks of dimers through the crystal. The 3-bromopropyl chain sits over the pyridine ring of the neighbouring molecule and the triazole rings of nearby molecules are adjacent [centroid-centroid distance = 4.691 (4) Å]. Unlike the dimer units, the extended stacks appear not to be stabilized by π-π interactions, the mean interplanar distance between dimers of 1 is 4.060 Å are outside the range normally expected for a π-π interaction. A steric interaction between the bromopropyl chain on one molecule of 1 and the pyridine ring on the adjacent molecule in the next dimer prevents a closer face to face interaction of the aromatic rings in the different dimer units.

### Experimental

The title compound, C_10_H_11_BrN_4_ (1) was obtained as a by-product during the attempted synthesis of a ditopic propyl bridged bis((2-pyridyl)-1,2,3-triazole) ligand. X-ray quality colourless crystals were obtained by slow evaporation of a petroleum ether solution of 1 but were weakly diffracting.

To a stirred solution of 1,3-dibromopropane (0.301 g, 1.5 mmol, 1.00 eq.) in DMF/H_2_O (15 ml, 4:1) was added NaN_3_ (0.205 g, 3.1 mmol, 2.05 eq.), Na_2_CO_3_ (0.13 g, 1.2 mmol, 0.8 eq.), CuSO_4_.5H_2_O (0.150 g, 0.6 mmol, 0.40 eq.), ascorbic acid (0.210 g, 1.2 mmol, 0.80 eq). 2-Ethynylpyridine (0.323 g, 3.1 mmol, 2.05 eq.) was added and the reaction mixture was stirred at room temperature for 20 h. The resulting suspension was then partitioned between aqueous NH_4_OH/edta (1 *M*, 100 ml) and CH_2_Cl_2_ (50 ml) and the layers separated. The organic phase was washed with H_2_O (100 ml) and brine (100 ml), dried (MgSO_4_) and the solvent removed under reduced pressure. Chromatography (gradient CH_2_Cl_2_/acetone to a ratio 9:1) gave the product as a white solid. Yield: 0.190 g, 47%. Mp 89–91 °C; ^1^H NMR (300 MHz, CDCl_3_) δ 8.58 (ddd, J = 0.9, J = 1.7, J = 4.9, 1H, H_1_), 8.23–8.12 (m, 2H, H_4,5_), 7.78 (td, J = 1.8, J = 7.8, 1H, H_3_), 7.26–7.21 (m, 1H, H_2_), 4.62 (t, J = 6.5, 2H, H_10_), 3.40 (t, J = 6.2, 2H, H_8_), 2.52 (p, J = 6.4, 2H, H_9_); ^13^C NMR (75 MHz, CDCl_3_) δ: 150.2, 149.5, 148.5, 136.9, 122.9, 122.6, 120.3, 48.3, 32.7, 29.3; I.*R*. (KBr): ν(cm^-1^) 3400–3200 (br), 3116, 3087, 2924, 1701, 1617, 1607, 1598, 1572, 1548, 1473, 1466, 1447, 1421, 1358, 1317, 1287, 1250, 1224, 1204, 1144, 1080, 1048, 983, 969, 891, 855, 844, 826, 786, 744, 708; HRESI-MS (MeOH): m/*z* = 289.0066 [*M*+Na]+ (calc. for C_10_H_11_^79^BrN_4_Na 289.0065 [*M*+Na]+), 291.0040 [*M*+Na]+ (calc. for C_10_H_11_^81^BrN_4_Na 291.0044 [*M*+Na]+; Anal. calcd for C_10_H_11_BrN_4_(H_2_O): C, 44.96; H, 4.15; N, 20.97; Found: C, 45.36; H, 4.24; N, 21.03.

### Refinement

All H-atoms bound to carbon were refined using a riding model with d(C—H) = 0.93 Å, *U*_iso_=1.2*U*_eq_ (C) for the CH H atoms and d(C—H) = 0.97 Å, *U*_iso_ = 1.2*U*_eq_ (C) for CH_2_ H atoms.

### Crystal data

**Table:**

C_10_H_11_BrN_4_	*Z* = 2
*M**_r_* = 267.14	*F*(000) = 268
Triclinic, *P*1	*D*_x_ = 1.608 Mg m^−^^3^
Hall symbol: -P 1	Melting point: 362 K
*a* = 5.658 (2) Å	Mo *K*α radiation, λ = 0.71073 Å
*b* = 9.688 (4) Å	Cell parameters from 5048 reflections
*c* = 10.191 (4) Å	θ = 3.1–33.3°
α = 84.498 (3)°	µ = 3.70 mm^−^^1^
β = 85.663 (2)°	*T* = 90 K
γ = 83.854 (2)°	Irregular, colourless
*V* = 551.6 (4) Å^3^	0.53 × 0.23 × 0.11 mm

### Data collection

**Table:**

Bruker APEXII CCD area-detector diffractometer	1879 independent reflections
Radiation source: fine-focus sealed tube	1759 reflections with *I* > 2σ(*I*)
graphite	*R*_int_ = 0.043
φ and ω scans	θ_max_ = 25.0°, θ_min_ = 4.0°
Absorption correction: multi-scan (*SADABS*; Bruker, 2004)	*h* = −6→6
*T*_min_ = 0.358, *T*_max_ = 0.66	*k* = −11→11
8776 measured reflections	*l* = −12→12

### Refinement

**Table:**

Refinement on *F*^2^	Primary atom site location: structure-invariant direct methods
Least-squares matrix: full	Secondary atom site location: difference Fourier map
*R*[*F*^2^ > 2σ(*F*^2^)] = 0.027	Hydrogen site location: inferred from neighbouring sites
*wR*(*F*^2^) = 0.071	H-atom parameters constrained
*S* = 0.97	*w* = 1/[σ^2^(*F*_o_^2^) + (0.0372*P*)^2^ + 0.5592*P*] where *P* = (*F*_o_^2^ + 2*F*_c_^2^)/3
1879 reflections	(Δ/σ)_max_ = 0.001
136 parameters	Δρ_max_ = 0.64 e Å^−^^3^
0 restraints	Δρ_min_ = −0.61 e Å^−^^3^

### Special details

**Table:**

Geometry. All e.s.d.'s (except the e.s.d. in the dihedral angle between two l.s. planes) are estimated using the full covariance matrix. The cell e.s.d.'s are taken into account individually in the estimation of e.s.d.'s in distances, angles and torsion angles; correlations between e.s.d.'s in cell parameters are only used when they are defined by crystal symmetry. An approximate (isotropic) treatment of cell e.s.d.'s is used for estimating e.s.d.'s involving l.s. planes.
Refinement. Refinement of *F*^2^ against ALL reflections. The weighted *R*-factor *wR* and goodness of fit *S* are based on *F*^2^, conventional *R*-factors *R* are based on *F*, with *F* set to zero for negative *F*^2^. The threshold expression of *F*^2^ > σ(*F*^2^) is used only for calculating *R*-factors(gt) *etc*. and is not relevant to the choice of reflections for refinement. *R*-factors based on *F*^2^ are statistically about twice as large as those based on *F*, and *R*- factors based on ALL data will be even larger.

### Fractional atomic coordinates and isotropic or equivalent isotropic displacement parameters (Å^2^)

**Table:**

	*x*	*y*	*z*	*U*_iso_*/*U*_eq_	
Br1	0.84285 (5)	−0.19395 (3)	0.97535 (3)	0.03839 (13)	
N3	0.3211 (3)	0.1602 (2)	0.6731 (2)	0.0243 (4)	
C6	0.4287 (4)	0.2879 (2)	0.4946 (2)	0.0177 (5)	
N2	0.2396 (4)	0.2401 (2)	0.5713 (2)	0.0236 (4)	
N1	0.6005 (4)	0.4000 (2)	0.2965 (2)	0.0230 (4)	
N4	0.5611 (3)	0.15753 (19)	0.66118 (19)	0.0188 (4)	
C5	0.3999 (4)	0.3838 (2)	0.3747 (2)	0.0179 (5)	
C7	0.6350 (4)	0.2341 (2)	0.5518 (2)	0.0185 (5)	
H7	0.7908	0.2476	0.5215	0.022*	
C4	0.1805 (4)	0.4545 (2)	0.3468 (2)	0.0216 (5)	
H4	0.0447	0.4385	0.4010	0.026*	
C8	0.7073 (4)	0.0790 (2)	0.7608 (2)	0.0212 (5)	
H8A	0.6075	0.0576	0.8403	0.025*	
H8B	0.8267	0.1360	0.7832	0.025*	
C2	0.3717 (5)	0.5674 (2)	0.1552 (2)	0.0244 (5)	
H2	0.3677	0.6302	0.0802	0.029*	
C1	0.5814 (4)	0.4892 (2)	0.1884 (2)	0.0249 (5)	
H1	0.7170	0.4993	0.1322	0.030*	
C9	0.8298 (5)	−0.0555 (3)	0.7124 (2)	0.0291 (6)	
H9A	0.7101	−0.1168	0.7002	0.035*	
H9B	0.9133	−0.0349	0.6273	0.035*	
C3	0.1681 (4)	0.5495 (2)	0.2364 (2)	0.0242 (5)	
H3	0.0245	0.6008	0.2172	0.029*	
C10	1.0035 (5)	−0.1294 (3)	0.8062 (3)	0.0309 (6)	
H10A	1.1188	−0.0667	0.8222	0.037*	
H10B	1.0890	−0.2087	0.7663	0.037*	

### Atomic displacement parameters (Å^2^)

**Table:**

	*U*^11^	*U*^22^	*U*^33^	*U*^12^	*U*^13^	*U*^23^
Br1	0.04089 (19)	0.03674 (19)	0.03195 (18)	0.00206 (12)	0.00069 (13)	0.01657 (12)
N3	0.0180 (10)	0.0273 (11)	0.0260 (11)	−0.0011 (8)	−0.0002 (9)	0.0033 (9)
C6	0.0187 (11)	0.0154 (10)	0.0191 (11)	−0.0011 (9)	−0.0003 (9)	−0.0032 (9)
N2	0.0191 (10)	0.0265 (10)	0.0241 (11)	−0.0024 (8)	−0.0010 (8)	0.0032 (9)
N1	0.0214 (10)	0.0240 (10)	0.0227 (10)	−0.0008 (8)	0.0001 (9)	−0.0013 (8)
N4	0.0171 (9)	0.0191 (9)	0.0194 (10)	0.0001 (8)	−0.0017 (8)	0.0001 (8)
C5	0.0187 (11)	0.0152 (10)	0.0200 (11)	−0.0024 (9)	−0.0012 (9)	−0.0028 (9)
C7	0.0159 (11)	0.0181 (10)	0.0212 (11)	−0.0013 (9)	−0.0002 (9)	−0.0014 (9)
C4	0.0170 (11)	0.0229 (11)	0.0246 (12)	−0.0011 (9)	−0.0006 (10)	−0.0021 (10)
C8	0.0240 (12)	0.0200 (11)	0.0187 (11)	0.0020 (9)	−0.0037 (10)	−0.0007 (9)
C2	0.0344 (14)	0.0183 (11)	0.0208 (12)	−0.0028 (10)	−0.0075 (11)	0.0015 (9)
C1	0.0251 (12)	0.0266 (12)	0.0220 (12)	−0.0039 (10)	0.0024 (10)	0.0015 (10)
C9	0.0424 (15)	0.0230 (12)	0.0198 (12)	0.0072 (11)	−0.0026 (11)	−0.0027 (10)
C3	0.0239 (12)	0.0204 (11)	0.0281 (13)	0.0029 (10)	−0.0067 (11)	−0.0030 (10)
C10	0.0357 (15)	0.0261 (13)	0.0264 (13)	0.0093 (11)	0.0030 (12)	0.0028 (10)

### Geometric parameters (Å, °)

**Table:**

Br1—C10	1.966 (3)	C8—C9	1.516 (3)
N3—N2	1.315 (3)	C8—H8A	0.9700
N3—N4	1.352 (3)	C8—H8B	0.9700
C6—N2	1.371 (3)	C2—C3	1.383 (4)
C6—C7	1.373 (3)	C2—C1	1.384 (4)
C6—C5	1.471 (3)	C2—H2	0.9300
N1—C1	1.337 (3)	C1—H1	0.9300
N1—C5	1.351 (3)	C9—C10	1.501 (4)
N4—C7	1.344 (3)	C9—H9A	0.9700
N4—C8	1.463 (3)	C9—H9B	0.9700
C5—C4	1.388 (3)	C3—H3	0.9300
C7—H7	0.9300	C10—H10A	0.9700
C4—C3	1.384 (3)	C10—H10B	0.9700
C4—H4	0.9300		
			
N2—N3—N4	106.83 (18)	H8A—C8—H8B	107.9
N2—C6—C7	108.4 (2)	C3—C2—C1	118.2 (2)
N2—C6—C5	122.9 (2)	C3—C2—H2	120.9
C7—C6—C5	128.7 (2)	C1—C2—H2	120.9
N3—N2—C6	108.78 (19)	N1—C1—C2	123.8 (2)
C1—N1—C5	117.1 (2)	N1—C1—H1	118.1
C7—N4—N3	111.53 (18)	C2—C1—H1	118.1
C7—N4—C8	127.7 (2)	C10—C9—C8	112.8 (2)
N3—N4—C8	120.73 (19)	C10—C9—H9A	109.0
N1—C5—C4	122.9 (2)	C8—C9—H9A	109.0
N1—C5—C6	115.7 (2)	C10—C9—H9B	109.0
C4—C5—C6	121.4 (2)	C8—C9—H9B	109.0
N4—C7—C6	104.5 (2)	H9A—C9—H9B	107.8
N4—C7—H7	127.8	C2—C3—C4	119.3 (2)
C6—C7—H7	127.8	C2—C3—H3	120.4
C3—C4—C5	118.6 (2)	C4—C3—H3	120.4
C3—C4—H4	120.7	C9—C10—Br1	111.7 (2)
C5—C4—H4	120.7	C9—C10—H10A	109.3
N4—C8—C9	111.80 (19)	Br1—C10—H10A	109.3
N4—C8—H8A	109.3	C9—C10—H10B	109.3
C9—C8—H8A	109.3	Br1—C10—H10B	109.3
N4—C8—H8B	109.3	H10A—C10—H10B	107.9
C9—C8—H8B	109.3		

### Hydrogen-bond geometry (Å, °)

**Table:**

*D*—H···*A*	*D*—H	H···*A*	*D*···*A*	*D*—H···*A*
C7—H7···N2^i^	0.93	2.62	3.449 (4)	149
C10—H10B···N1^ii^	0.97	2.51	3.450 (4)	164

Symmetry codes: (i) *x*+1, *y*, *z*; (ii) −*x*+2, −*y*, −*z*+1.
